# Curriculum-Based Measurement progress data: Effects of graph pattern on ease of interpretation

**DOI:** 10.1007/s11618-018-0836-9

**Published:** 2018-07-02

**Authors:** Christine A. Espin, Nadira Saab, Ron Pat-El, Priscilla D. M. Boender, Joost van der Veen

**Affiliations:** 1 Department of Education and Child Studies, Leiden University, Wassenaarseweg 52, 2333 AK Leiden, The Netherlands; 2 Department of Methods and Statistics, The Open University of the Netherlands, Postbus 2960, 6401 DL Heerlen, The Netherlands; 3 RAET Academy, Amersfoort, The Netherlands

**Keywords:** Curriculum-Based Measurement, Graph interpretation, Graph comprehension, Formative assessment, Progress monitoring, Learning disabilities, Curriculum-Based Measurement, Interpretation von Diagrammen, Verstehen von Diagrammen, Formatives Assessment, Lernverlaufsdiagnostik, Lernschwierigkeiten

## Abstract

Curriculum-Based Measurement (CBM) is a system for monitoring the progress of and evaluating instructional program effectiveness for students with learning difficulties. Although a large amount of research has been conducted on CBM, little has focused on the interpretation and use of the data for instructional decision-making, despite the fact that it is data use that leads to performance gains. In this study, we examine factors affecting the interpretation of CBM data. Specifically, we examine the effect of CBM graph patterns on ease of graph interpretation. Thirty college/university students completed a two-part study in which they viewed various slope-to-goal and slope-to-slope patterns of CBM-graphed data and answered decision-making questions. Response times and accuracy were measured. Results revealed that graph patterns differed in terms of ease of interpretation. Differences depended on the type of question. Implications for interpretation and use of CBM data for decision-making are discussed.

**Electronic supplementary material:**

The online version of this article (10.1007/s11618-018-0836-9) contains supplementary material, which is available to authorized users.

## Zusammenfassung

Mit dem Ansatz des Curriculum-Based Measurement (CBM) können Lernverläufe überwacht und die Wirksamkeit instruktionaler Programme für Schülerinnen und Schüler mit Lernschwierigkeiten geprüft werden. Wenngleich viele Studien zu dem CBM Ansatz vorliegen, wurde dem Aspekt der Interpretation und Nutzung der Daten für pädagogische Entscheidungen bislang wenig Beachtung geschenkt, obwohl die Nutzung von CBM-Daten zu Leistungsverbesserungen führt. In dieser Studie werden Merkmale von Lernverlaufsgraphen daraufhin geprüft, in welchem Maße sie die Interpretierbarkeit von CBM-Daten beeinflussen. Dreißig Studierende bearbeiteten mehrere CBM Verlaufsmuster, bei denen Variationen hinsichtlich der Entwicklung von Lernverläufen zu zwei Zeitpunkten und hinsichtlich der Annäherung von Lernverläufen im Hinblick auf Lernziele vorgenommen wurden. Von den Studierenden mussten jeweils pädagogische Entscheidungen getroffen werden, wobei Reaktionszeiten und Akkuratheit der Antworten gemessen wurden. Es zeigte sich, dass Merkmale der Verlaufsmuster einen Einfluss auf deren Interpretierbarkeit haben. Unterschiede ergeben sich vor allem im Hinblick auf die Art der pädagogischen Entscheidungen. Implikationen im Hinblick auf die Interpretation und die Nutzung von CBM-Daten für pädagogische Entscheidungen werden diskutiert.

## Introduction

Curriculum-based Measurement (CBM) is a method for closely monitoring the progress of students with learning difficulties, and for evaluating the effectiveness of their instructional programs (Deno 1985; Deno and Fuchs 1987). CBM data are collected on a frequent basis (e. g., weekly) using tasks representative of general performance and progress in academic domains. For example, in CBM reading, students read aloud from a text passage, and the number of words read correctly in 1 min is counted, or students read silently from a maze passage, and the number of correct selections in 2 min is counted. (A maze is a text in which every seventh word is deleted and replaced with the correct word and two distractors).

**Fig. 1:**
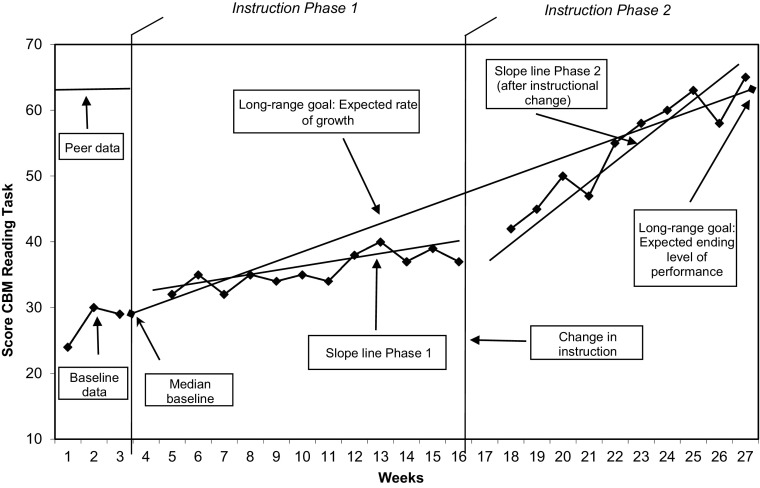
Sample Curriculum-Based Measurement (CBM) progress graph

Scores from CBM tasks are placed on a graph that visually displays the student’s progress across the school year (see Fig. 1). The graph includes: (1) baseline data, representing the student’s beginning level of performance, and a line displaying the peer level of performance during the baseline period; (2) a long-range goal, representing the expected (desired) ending level of performance and the expected rate of growth; (3) data points, representing the student’s scores on the CBM measures; (4) slope lines, representing the student’s actual rate of growth within each instructional phase. The graph guides the teacher’s instructional decision-making. Thus, if the slope line is less steep than or below the goal line, it signals an *ineffective *instructional program, and a need to change the student’s instruction (see Fig. 1, Phase 1). If the slope line is steeper than and above the goal line, it signals an *effective *instructional program (see Fig. 1, Phase 2) and instruction continues as it is. If the slope line is *much* steeper than the goal line (as is the case in Fig. 1, Phase 2), the goal is raised. The graph presented in Fig. 1 includes only two phases of instruction. An actual CBM graph might include four or five different phases of instruction across the school year.

Although a large body of research has accumulated on CBM, the majority of the research has focused on the psychometric properties of the measures used within CBM (see reviews Marston 1989; Wayman et al. 2007; Reschly et al. 2009; Espin et al. 2017a), with less research focused on the interpretation and use of the data. An exception to this is research carried out by L. Fuchs, D. Fuchs and colleagues in the 1980s and 1990s, which focused on the use of computer software to help teachers collect, score, and graph CBM data, and to make effective instructional decisions (see Fuchs and Fuchs 1989, 2002; Fuchs et al. 1989, 1994, 2003; Stecker et al. 2005). The software provided teachers with prompts for raising the goal or changing instruction, and with diagnostic and expert-systems analysis to aid decision-making about what and how to change instruction (see reviews Fuchs et al. 1994, 2003; Fuchs and Fuchs 2002; Stecker et al. 2005).

The research of Fuchs, Fuchs, and colleagues demonstrated that when teachers responded to CBM data by modifying instructional programs and raising the goals, student achievement improved significantly, but that teachers needed assistance in responding to the data (Fuchs et al. 1994; Stecker et al. 2005). The computer software provided teachers with the needed assistance and improved teachers’ response to the data and their subsequent use of the data for instructional planning, which, in turn, improved student achievement (Fuchs et al. 1994, 2003; Fuchs and Fuchs 2002; Stecker et al. 2005). What was not examined in the research was why teachers had difficulty responding to the CBM data in the first place.

There are likely many reasons for teachers’ difficulties in responding to CBM data, but one potential reason is teachers’ ability to read and interpret the CBM graphs. One might assume that it is not necessary for teachers to read and interpret the CBM progress graphs; after all, electronic progress-monitoring programs can provide prompts and instructional recommendations to teachers. However, such an assumption may not be warranted. First, research by Fuchs, Fuchs, and colleagues demonstrated that the teacher is an essential part of the decision-making process. Active involvement of the teachers in decision-making enhanced the effects of computer supports (Fuchs and Fuchs 1989; Fuchs et al. 1989). Second, and somewhat related to the first, teachers may not trust the instructional recommendations being offered if they do not understand the data. For example, research has demonstrated that teachers vary in their beliefs about the meaning and usefulness of data in instructional decision-making, and are somewhat wary about the trustworthiness and utility of data (Foegen et al. 2001; Landrum et al. 2007). A better understanding of the data might lead to better use of the data for decision-making. Third, and most directly related to the research presented here, graph interpretation is not necessarily easy, but is a “complex and challenging activity” (Glazer 2011, p. 183).

### CBM graph interpretation/comprehension

The ability to accurately read and interpret graphed data is referred to as *graph comprehension* (Friel et al. 2001; Shah and Hoeffner 2002). Graph comprehension is depicted as occurring at three levels (Curcio 1987; Friel et al. 2001): *reading the data* (extracting data from the graph), *reading between the data* (interpreting relationships between the data in the graph), and *reading beyond the data *(extrapolating from data in the graph to make inferences or predictions). (See applications of this framework in Galesic and Garcia-Retamero 2011; Boote 2014; Kim et al. 2014) Van den Bosch et al. (2017) applied this framework to CBM, and conceptualized CBM graph comprehension as consisting of: (1) *reading the data, *describing the scores and slope lines on the graph; (2) *reading between the data, *interpreting the relations between different graph components such as the slope and goal lines, and, (3) *reading beyond the data, *linking the progress data to the students’ instruction.

In recent years, there has been a series of studies focused on CBM graph comprehension. These studies have demonstrated that both inservice (Espin et al. 2017b) and preservice teachers (Wagner et al. 2017) have difficulty with CBM graph comprehension, and that years of experience with CBM does not relate to CBM graph comprehension (Espin et al. 2017b). Furthermore, the research has suggested that teachers’ difficulties are not due solely to a lack of general graph-reading skills (van den Bosch et al. 2017; see also Zeuch et al. 2017): Even statistics- and educational-assessment experts with several years of experience in graph interpretation had some difficulty comprehending CBM graphs (van den Bosch et al. 2017). Finally, the research has demonstrated that teachers’ experience the greatest difficulty with *reading between *and *reading beyond* the data (van den Bosch et al. 2017); that is, teachers have difficulty interpreting relations between the graph components and then linking the information to instruction.

The findings of the research on CBM graph comprehension suggest that it is worthwhile to consider, among other questions, what makes CBM graphs difficult to interpret. If even statistics- and educational-assessment experts have difficulty interpreting CBM graphs, it is not merely a matter of improving general graph interpretation. It is more likely the case that teachers need specific training and instruction in interpreting CBM graphs. As a part of the training, it would be useful to know which specific aspects of the CBM graphs are difficult to interpret.

### Interpretation of CBM graph patterns

Research on general graph reading demonstrates the need to consider graph-related factors in studying graph comprehension. Specific to the current research, the visual characteristics of a graph can influence the reader’s ability to comprehend the graph (Friel et al. 2001; Shah and Hoeffner 2002). For example, Trickett and Trafton (2004, 2006) studied the interpretation of line graphs and found that the more spatial transformation required to interpret the graph, the more difficult the graph was to interpret (Trickett and Trafton 2004, 2006). *Spatial transformation* refers to the need for the viewer to mentally create or delete something on a graph in order to understand the graph. Trickett and Trafton (2004) asked participants to mentally extend a line in order to predict what the value of the line would be at the end point of the graph. The distance that the line needed to be mentally extended was varied. The greater the distance for mentally extending the line—that is, the more spatial transformation required—the longer the response time and the lower the accuracy of the participants’ responses. The results of Trickett and Trafton (2004) demonstrated that even simple line graphs (for example, CBM graphs) can be difficult to interpret, especially if the graph patterns require spatial transformations in order to correctly interpret them.

In the current study, we examine the extent to which different CBM graph patterns are easy or difficult to interpret. Patterns that are identified as difficult to interpret can be singled out in the future for focused instruction in CBM training and/or augmented with graphic aides in CBM graphing programs.

### Purpose and research questions

The purpose of the current study was to examine participants’ ability to interpret various graph patterns found in CBM progress graphs. Participants viewed a number of different CBM graph patterns, and for each pattern, answered a series of instructional decision-making questions.

Graph interpretation was measured via response time and accuracy for each question. We included both response times and accuracy because each reflected ease of graph-pattern interpretation in slightly different ways. For example, although we might expect response times and accuracy to co-vary for difficult patterns—with longer response times being associated with lower accuracy—this might not always be the case. In some cases, response times might be short and accuracy low, indicating that a pattern is easy to ***mis***interpret; that is, that respondents think they know the answer, but the answers are incorrect. Conversely, response times might be long and accuracy high, indicating that a pattern is difficult to interpret, but with careful inspection, the answer becomes clear.

Both longer response times and lower accuracy are potentially problematic in practice. If teachers have to exert extra effort to interpret CBM graphs, they may be less likely to inspect the graphs and use the data for decision-making. Likewise, if teachers incorrectly interpret the graphs, they may make incorrect instructional decisions. By including both response times and accuracy, we cast a wide net for identifying potentially problematic graph patterns.

One research question was addressed in the study: *Are there differences in response times and accuracy rates when answering instructional decision-making questions about various CBM graph patterns? *We hypothesized that ease of interpretation would vary with graph pattern and, based on the work of Trickett and Trafton (2004), that the more spatial transformation required, the more difficult the interpretation of the pattern would be. In this study, the focus was on instructional decisions related to changing instruction (but not raising the goal). In addition, because our focus was on the graph itself rather than on the graph reader, we included participants with little to no knowledge of or background in CBM.

## Method

### Participants

Participants were 30 college/university students (22 female, 8 male) from the Netherlands. Participants were recruited via an undergraduate research experience program as well as word of mouth. Ages ranged from 19 to 56 years (*M* = 24.75, *SD* = 7.48). Twenty-five (83.3%) of the students had taken courses in statistics at university and seven (23.3%) were familiar with CBM graphs. None of the participants had ever implemented CBM. There were two parts to the study (see next section). All students participated in both parts of the study.

### Study: Parts 1 and 2

Parts 1 and 2 of the study focused on the two types of decisions made in interpreting CBM graphs. Part 1 focused on *slope-to-goal *decisions, which involved comparing the student’s actual rate of growth (slope) to the expected rate of growth (goal) to determine whether the intervention had a positive effect and whether the student would reach the long-range goal. Part 2 focused on *slope-to-slope *decisions, which involved comparing the slope *after* an instructional change to the slope *before* the instructional change to determine whether the change was effective.

### Materials and procedure

Before beginning the experiment, participants completed a demographic questionnaire. Graphs with accompanying questions were then presented via E‑Prime, a computer program that allows for measurement of response times. For each part of the study, participants were first given detailed instructions (see Appendix A), after which they completed five practice trials to ensure that they understood the task. Participants could ask the researcher questions while completing the five practice trials. The practice trials were presented in a fixed order. If participants made a mistake during the practice trials, the researcher provided corrective feedback. All participants completed at least four of the five practice trials correctly, and all reported that they understood the task.

After the practice trials, participants completed the experimental trials. Each experimental trial began with a moment of fixation (a plus sign presented in the middle of the computer screen), followed by the presentation of a graph and the corresponding question/s. Participants pressed a key to respond. After participants responded, a second moment of fixation occurred, and then the next graph and question/s were presented. All participants completed Part 1 and then Part 2 of the study. The entire session, including both Parts 1 and 2, lasted approximately 30 min.

### Design and independent variables

**Fig. 2:**
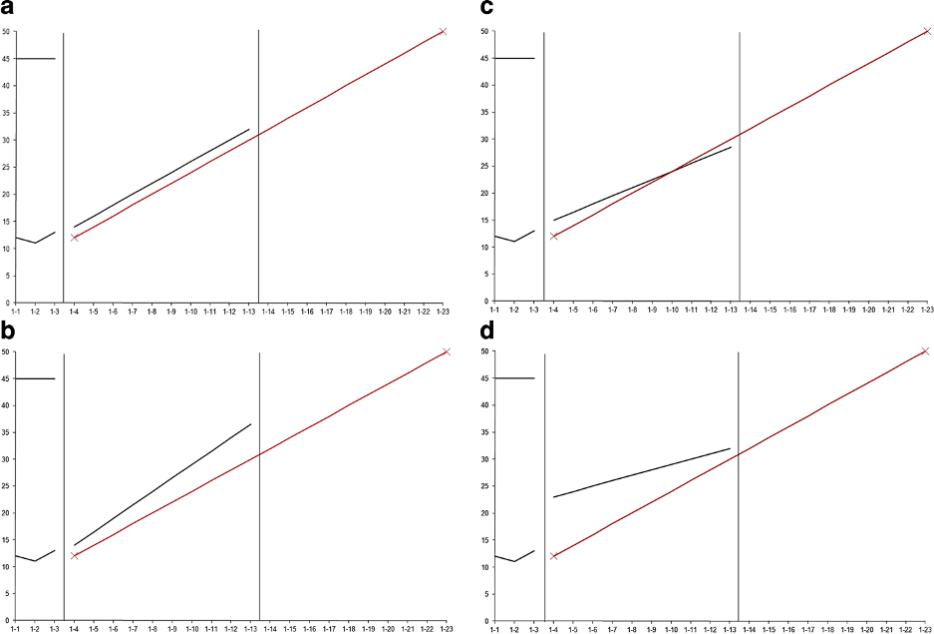
Sample slope-to-goal comparison patterns with slope line beginning above goal line: Parallel (**a**), Divergent (**b**), Crossed (**c**), and Convergent (**d**). Similar patterns were presented with slope line beginning below goal line. Answers to the three decision-making questions, (1) *Will the student reach the goal? *(2) *Did the instruction have a* *positive effect?* (3) *Should the teacher change the instruction?,* are as follows: **a** (yes, yes, no); **b** (yes, yes, no); **c** (no, yes, yes); **d** (no, yes, yes)

*Part 1: Slope-to-goal comparison. *Part 1 was a multivariate 4 × 2 within-subjects factorial design. In Part 1, the slope-to-goal pattern and slope start point were manipulated (see Fig. 2 for examples). For *pattern, *the position of the slope line relative to the goal line was varied in four conditions: (1) parallel, (2) crossed, (3) divergent, and (4) convergent. For the *start point, *where the slope line began relative to the goal line varied in two conditions: (1) above; (2) below. Three examples were provided for each combination for a total of 24 graphs.

**Fig. 3:**
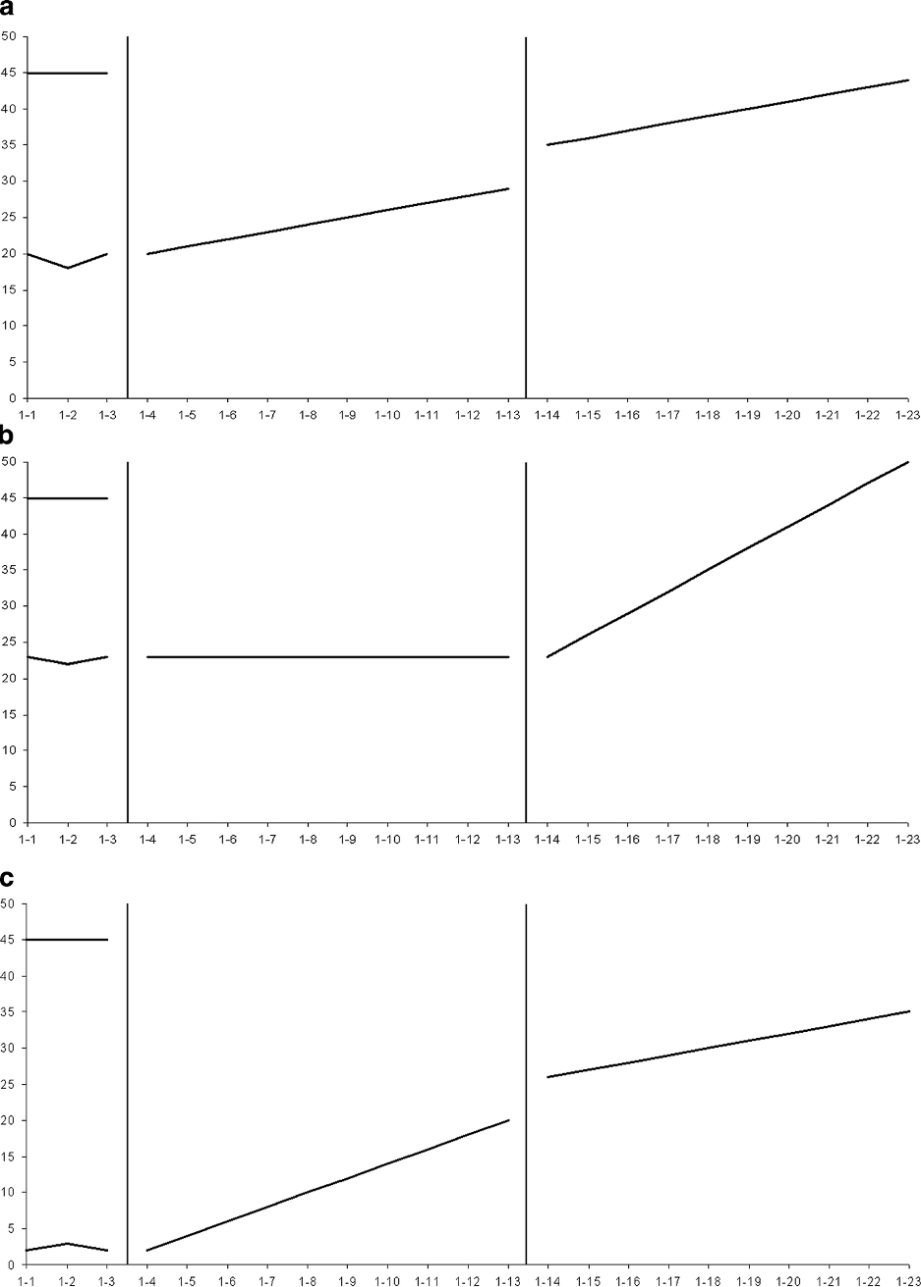
Sample slope-to-slope comparison patterns with slope lines in flat/positive direction: No difference or parallel with shift (**a**); large difference with no shift (**b**); small difference with shift (3c). Similar patterns were presented with slope lines in flat/negative directions. Answers to the decision-making question, *Was the change in instruction effective, *are as follows: **a** (yes); **b** (yes); **c** (no)

*Part 2: Slope-to-slope comparison. *Part 2 was a multivariate 3 × 2 × 2 within-subjects factorial design. In Part 2, shift, slope difference, and direction were manipulated (see Fig. 3 for examples). For *shift*, where the second slope began relative to the first was varied in two conditions: (1) no shift—second slope line began where the first slope line ended, and (2) shift—second slope line began above or below where the first slope line ended. For *difference,* differences in slope values, representing the student’s rate of growth in each instructional condition, were varied in three conditions: (1) large slope-value difference; (2) small slope-value difference; (3) no slope-value difference (parallel). Finally, the direction of the slopes was varied in two conditions: (1) positive—first slope was flat or positive and second slope was positive; (2) negative—first slope was flat or negative and second slope was negative. Three examples were provided for each combination for a total of 36 graphs.

### Dependent variables

Dependent variables were response times and accuracy. Response time was the number of milliseconds it took for the participants to answer each question. Accuracy was measured by counting the number of correct responses to decision-making questions for each graph condition. Response times and accuracy were recorded via E‑Prime.

For Part 1 of the study, slope-to-goal comparisons, decision-making questions were: (1) *Will the student reach the goal? *(2) *Did the instruction have a positive effect?* (3) *Should the teacher change the instruction?* The participant answered the question by pressing ‘i’ for ‘yes’, or ‘o’ for ‘no’. The order in which the graphs were presented was randomized, but participants completed the graphs in the same order. (Because there were multiple questions, graph order could not be varied across individuals in E‑Prime.) For Part 2 of the study, slope-to-slope comparisons, only one decision-making question was asked: *Was the change in instruction effective?* The participant could answer the question by pressing ‘i’ for ‘yes’, ‘o’ for ‘no’, and ‘p’ for ‘no difference.’ The order in which graphs were presented was randomized; participants completed the graphs in different random orders.

Correct answers to the questions were determined by the lead researcher, who had over 25 years of experience in conducting CBM research and giving CBM inservice/preservice trainings or university courses. (See Figs. 2 and 3 for the answers to the questions for the displayed graph patterns.) For some questions, the answer might be ambiguous because there are two potentially “correct” answers. We return to this point later in the discussion, but for purposes of this study, one answer was selected as the correct answer based on the researchers’ knowledge of the CBM literature and of the use of CBM in practice.

## Results

Paired t‑tests were performed per condition to test for differences in response times between correct and incorrect responses. A Bonferroni-correction was used to correct for multiple testing. Results revealed no significant differences in response times between correct and incorrect responses; thus, response times for correct and incorrect responses were aggregated.

### Part 1: Slope-to-goal comparisons

Data for Part 1 were inspected for missing values and univariate outliers. One univariate outlier (*z* > 3.00) was found in the parallel/above condition, where the response time for one participant was nearly three times longer than that of the next highest participant in that condition. In order to reduce the impact of the outlying response time score, the participant’s score was transformed to the second highest participants’ score in that condition. There were no missing values. Subsequent analyses were performed separately by question.

#### Question 1: *Will the student reach the goal?*

A repeated measures MANOVA was performed with slope pattern and start point as within subject factors and response time and accuracy as dependent variables. Assumptions of sphericity were violated for pattern (χ^2^_RT_(5) = 105.87, χ^2^_Acc_(5) = 27.70, *p* < 0.001) and for the interaction pattern x start point (χ^2^_RT_(5) = 62.77, *p* < 0.001, χ^2^_Acc_(5) = 12.69, *p* = 0.03), therefore degrees of freedom were corrected using Huyn-Feldt estimates of sphericity.

**Fig. 4:**
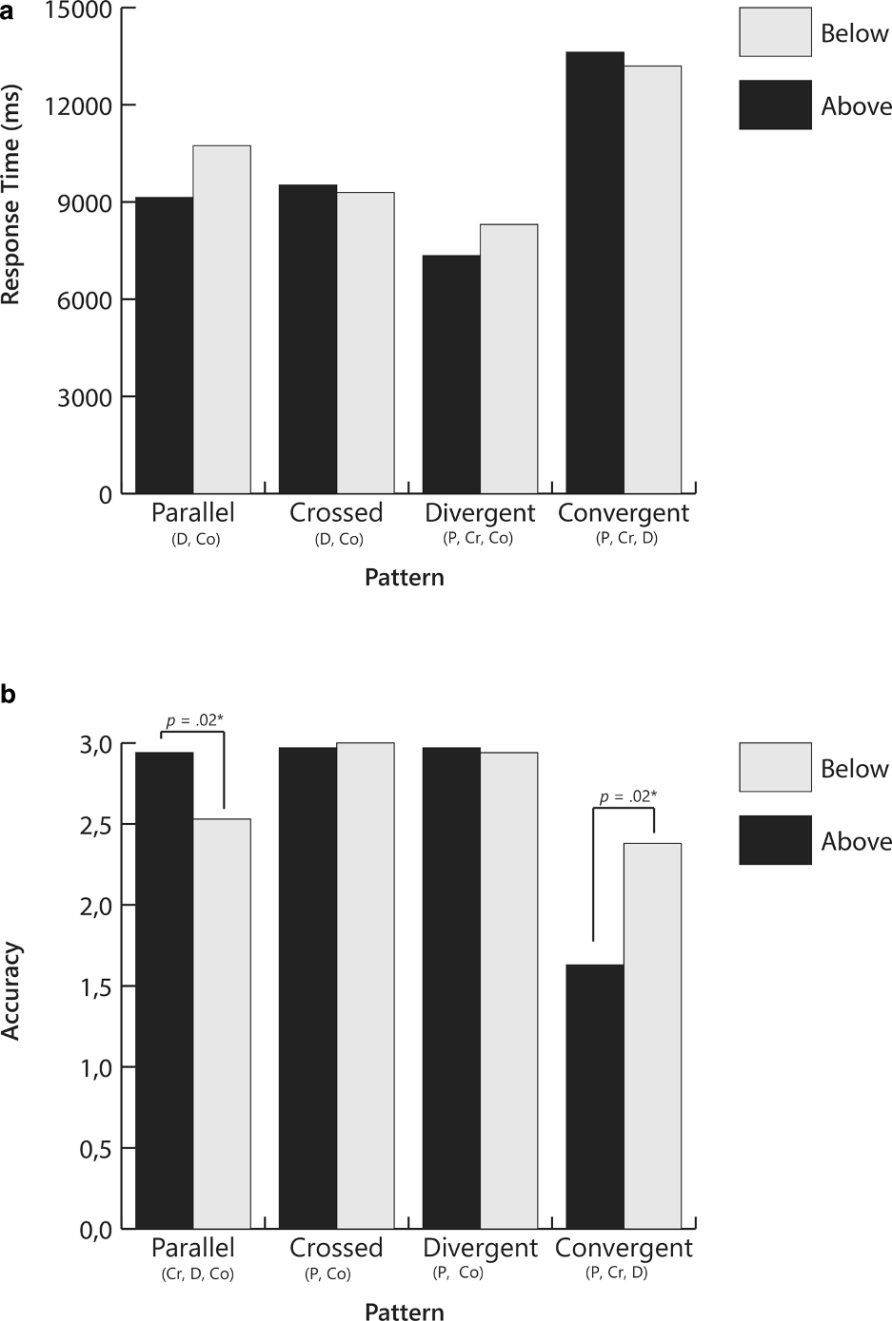
Mean response times and accuracy for slope-to-goal comparisons across pattern and start point for Question 1: *Will the student reach the goal?* Letters below bars signify significant differences between patterns. *P* Parallel; *Cr* Crossed; *D* Divergent; *Co* Convergent. *P-values* signify differences between start points within pattern

Results of the analysis are presented in Table 1. For ease of interpretation, results are also displayed in a bar graph in Fig. 4. Both response time and accuracy were affected by pattern (*V* = 0.60, *F*(6,174) = 12.41, *p* < 0.001, partial η^2^ = 0.30) with a stronger effect seen for accuracy (*F*(1.4,40.8) = 32.75, *p* < 0.001, partial η^2^ = 0.53) than for response time (*F*(1.7,48.2) = 9.47, *p* = 0.001, partial η^2^ = 0.25). There was no significant multivariate main effect for start point (*V* = 0.08, *F*(2,28) = 1.22, *p* = 0.31, partial η^2^ = 0.08). There was a significant interaction effect for pattern x start point (*V* = 0.26, *F*(6,174) = 4.31, *p* < 0.001, partial η^2^ = 0.13). The interaction effect was significant for accuracy (*F*(1.4,41.6) = 8.43, *p* = 0.002, partial η^2^ = 0.23), but not for response time (*F*(2.6,47.0) = 2.03, *p* = 0.12, partial η^2^  = 0.07).

**Table 1:** Means and Standard Errors for Response Time and Accuracy Across Slope Position and Start Points for Slope-to-Goal Comparison Questions

	Response Time				Accuracy			
Start point	Above		Below		Above		Below	
Pattern	M	SE	M	SE	M	SE	M	SE
*Question 1: Will the student reach the goal?*								
Parallel	9138.31	552.83	10,740.38	916.81	2.94	0.04	2.53	0.14
Crossed	9524.19	671.21	9293.00	754.61	2.97	0.03	3.00	0.00
Divergent	7346.41	433.35	8308.84	372.03	2.97	0.03	2.94	0.06
Convergent	13,626.22	1226.57	13,196.75	1384.23	1.63	0.19	2.38	0.18
*Question 2: Did the instruction have a positive effect?*								
Parallel	7570.91	675.89	8017.66	906.54	2.84	0.10	2.47	0.19
Crossed	9817.56	1020.64	6954.19	641.01	2.44	0.15	3.00	0.00
Divergent	7277.09	910.69	8006.59	759.24	2.94	0.06	2.41	0.21
Convergent	7659.09	687.03	6893.84	556.66	2.72	0.14	2.78	0.13
*Question 3: Should the teacher change the instruction?*								
Parallel	5815.09	432.64	6492.50	522.06	2.84	0.07	2.47	0.13
Crossed	7842.94	809.91	6041.56	541.83	2.69	0.10	2.88	0.07
Divergent	5972.75	483.80	8101.94	1037.86	2.97	0.03	2.69	0.12
Convergent	6287.59	601.56	7546.25	635.54	1.78	0.19	2.28	0.19

Response times reported in milliseconds; Accuracy is the number correct out of 3

*M* Mean; *SE* Standard error

Post-hoc tests with Bonferroni corrections revealed that, regardless of whether the start point was above or below the goal line, response times were significantly longer for convergent patterns than for parallel, crossed, or divergent patterns, and longer for the parallel and crossed patterns than for the divergent patterns. (Differences are noted in Fig. 3 with letters below the name of each condition.) Similarly, accuracy was lower for convergent patterns than for parallel, crossed, or divergent patterns, and lower for parallel patterns than for crossed or divergent patterns. Start point affected accuracy only, and then only for parallel and convergent patterns. For parallel patterns, accuracy was lower when the slope line began below the goal line, but for convergent patterns, it was lower when the slope line began above the goal line.

In sum, with regard to answering the question, *Will the student reach the goal?*, results revealed that the graph pattern that was the most difficult to interpret was one in which the slope lines converged upon the goal line. These patterns appeared to be especially difficult when the slope lines began above and converged upon the goal line. In contrast, the graph pattern that appeared to be the easiest to interpret was one in which the slope lines diverged from the goal line. Parallel patterns were difficult to interpret when the slope line began below the goal line.

#### Question 2: *Did the instruction have a positive effect?*

A repeated measures MANOVA was performed with pattern and start point as within subject factors and response time and accuracy as dependent variables. Assumptions of sphericity were violated for accuracy within pattern (χ^2^_Acc_(5) = 23.04, *p* < 0.001) and for the interaction pattern x start point (χ^2^_Acc_(5) = 65.04, *p* < 0.001), therefore degrees of freedom for accuracy were corrected using Huyn-Feldt estimates of sphericity.

**Fig. 5:**
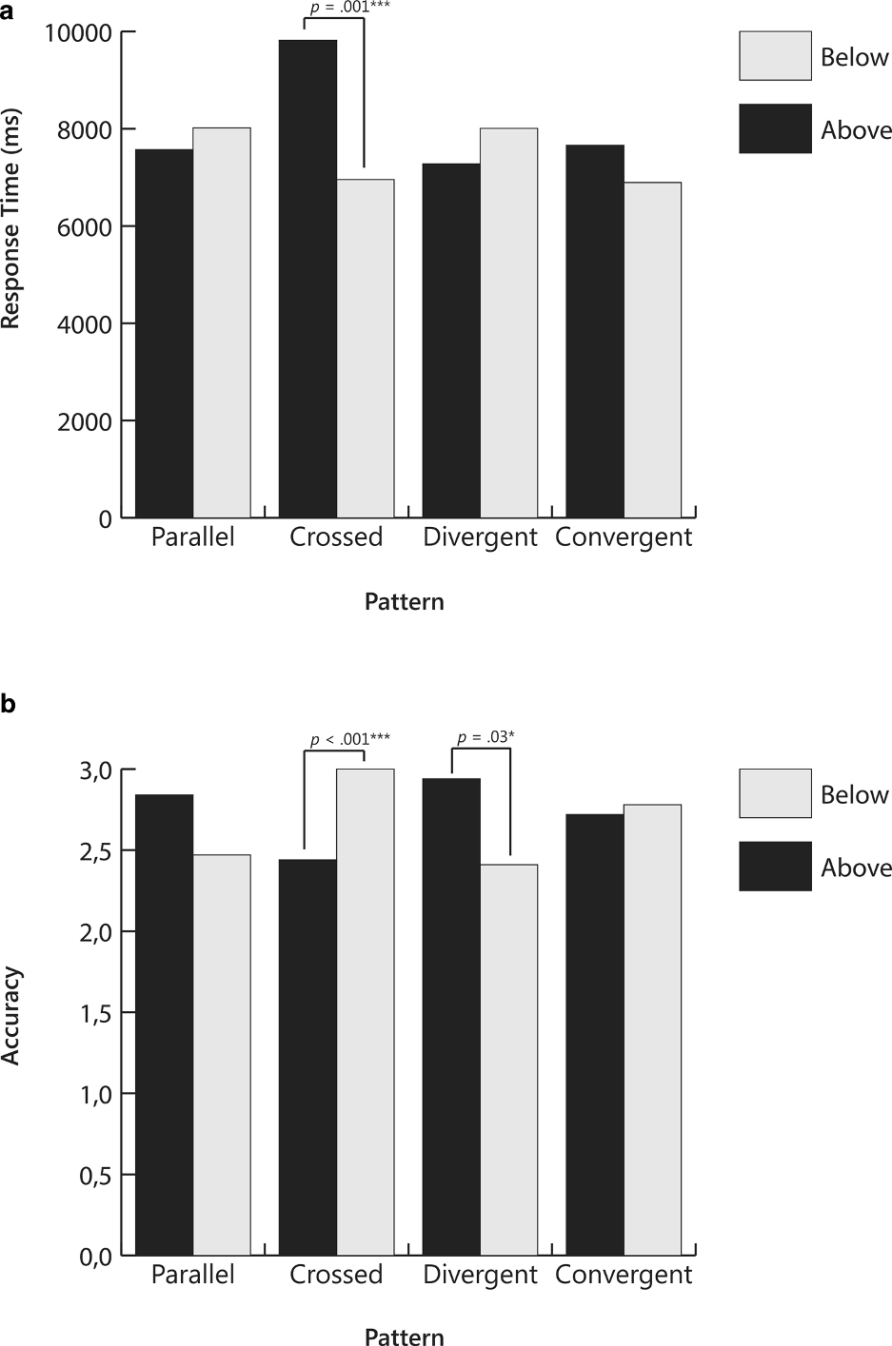
Mean response times and accuracy for slope-to-goal comparisons across pattern and start point for Question 2: *Did the instruction have a* *positive effect?* Letters below bars signify significant differences between patterns. *P* Parallel; *Cr* Crossed; *D* Divergent; *Co* Convergent. *P-values* signify differences between start points within pattern

Results of the analysis are presented in Table 1 and displayed in Fig. 5. There were no significant multivariate main effects for pattern (*V* = 0.07, *F*(6,186) = 1.22, *p* = 0.31, partial η^2^ = 0.04) or for start point (*V* = 0.09, *F*(2,30) = 1.45, *p* = 0.25, partial η^2^ = 0.09). There was a significant interaction effect pattern × start point (*V* = 0.26, *F*(6,186) = 4.63, *p* < 0.001, partial η^2^ = 0.13). The interaction effect was significant for both accuracy (*F*(1.6,48.3) = 8.79, *p* = 0.001, partial η^2^ = 0.22) and response time (*F*(3,93) = 4.32, *p* = 0.007, partial η^2^  = 0.12).

Post-hoc tests with Bonferroni corrections revealed that in the crossed condition, response time was longer and accuracy lower when the start point was above the goal line than when it was below the goal line. In the divergent condition, the reverse effect was found, but only for accuracy. Accuracy was lower when the start point was below the goal line than when it was above the goal line.

In sum, when answering the question, *Did the instruction have a positive effect?*, pattern did not influence interpretation, but start point did, especially for the crossed patterns where both response times and accuracy were affected. For divergent patterns, only accuracy was affected.

#### Question 3: *Should the teacher change the instruction?*

A repeated measures MANOVA was performed with pattern and start point as within subject factors and response time and accuracy as dependent variables. Assumptions of sphericity were violated for accuracy within pattern (χ^2^_Acc_(5) = 30.33, *p* < 0.001) and for the interaction pattern x start point (χ^2^_RT_(5) = 41.82, *p* < 0.001, χ^2^_Acc_(5) = 30.95, *p* < 0.001), therefore degrees of freedom were corrected using Huyn-Feldt estimates of sphericity.

**Fig. 6:**
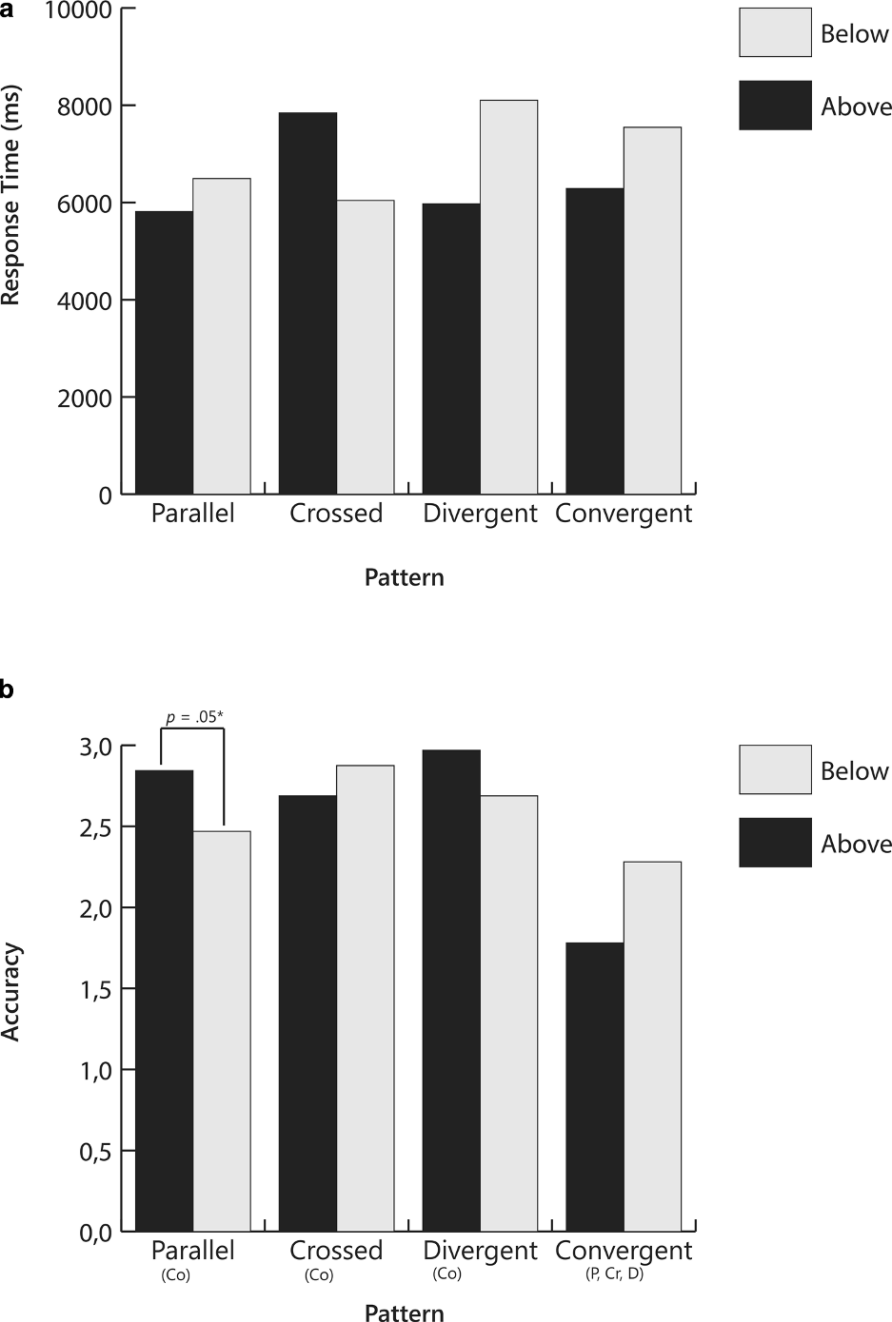
Mean response times and accuracy for slope-to-goal comparisons across pattern and start point for Question 3: *Should the teacher change the instruction?* Letters below bars signify significant differences between patterns. *P* Parallel; *Cr* Crossed; *D* Divergent; *Co* Convergent. *P-values* signify differences between start points within pattern

Results of the analysis are presented in Table 1 and displayed in Fig. 6. There was a significant main effect for pattern (*V* = 0.44, *F*(6,186) = 8.74, *p* < 0.001, partial η^2^ = 0.22), but the univariate effect was only significant for accuracy, (*F*(1.9,58.1) = 20.56, *p* < 0.001, partial η^2^ = 0.40) and not for response time (*F*(3,93) = 1.26, *p* = 0.294, partial η^2^ = 0.04). There was no significant multivariate main effect for start point (*V* = 0.07, *F*(2,30) = 1.22, *p* = 0.33, partial η^2^ = 0.07). There was a significant interaction effect between pattern and start point (*V* = 0.22, *F*(6,186) = 3.81, *p* = 0.001, partial η^2^ = 0.11), but the interaction effect was only significant for accuracy (*F*(1.8,55.1) = 5.16, *p* = 0.011, partial η^2^ = 0.14) and not for response time (*F*(1.6,58.6) = 3.02, *p* = 0.06, partial η^2^  = 0.09).

Post-hoc tests with Bonferroni corrections revealed that accuracy for convergent graph patterns was significantly lower than for all other graph patterns. Further, within the parallel condition, accuracy was significantly lower when the start point was below the goal line than when it was above the goal line.

In sum, when answering the question, *Should the teacher change the instruction?* convergent patterns proved once again to be difficult to interpret. With the exception of parallel patterns, start point had little effect. Before describing the results for Part 2 of the study, it is worthwhile to note that response times tended to decrease across the three questions. For example, for convergent patterns, means response times were approximately 13,600 ms (above) and 13,200 ms (below) for Question 1 compared to 6300 ms (above) and 7550 ms (below) for Question 3.

### Part 2: Slope-to-slope comparisons

In Part 2, participants were asked, *Was the change in instruction effective? *The data were inspected for missing values and univariate outliers. Two univariate outliers (*z* > 3.00) were found for the same participant in the no slope/unequal slope shift condition for both positive and negative slopes, where the participant’s response times were more than 2.5 times longer than that of other participants in those particular conditions. In order to reduce the impact of the outlying response times, the participant’s scores were transformed to the second highest participants’ score in those conditions. There were no missing values.

A Repeated Measures MANOVA was performed with shift (no shift/shift), slope difference (large/small/parallel), and direction (negative/positive) as within subject variables and response time and accuracy as dependent variables. Assumptions of sphericity were violated for slope difference (χ^2^_RT_(2) = 10.10, *p* = 0.006; χ^2^_Acc_(2) = 29.45, *p* < 0.001) and the interactions difference x direction (χ^2^_RT_(2) = 7.82, *p* = 0.02, χ^2^_Acc_(2) = 40.32, *p* < 0.001), difference x shift (χ^2^_Acc_(2) = 35.82, *p* < 0.001) and direction x difference x shift (χ^2^_Acc_(2) = 7.49, *p* = 0.024).

**Fig. 7:**
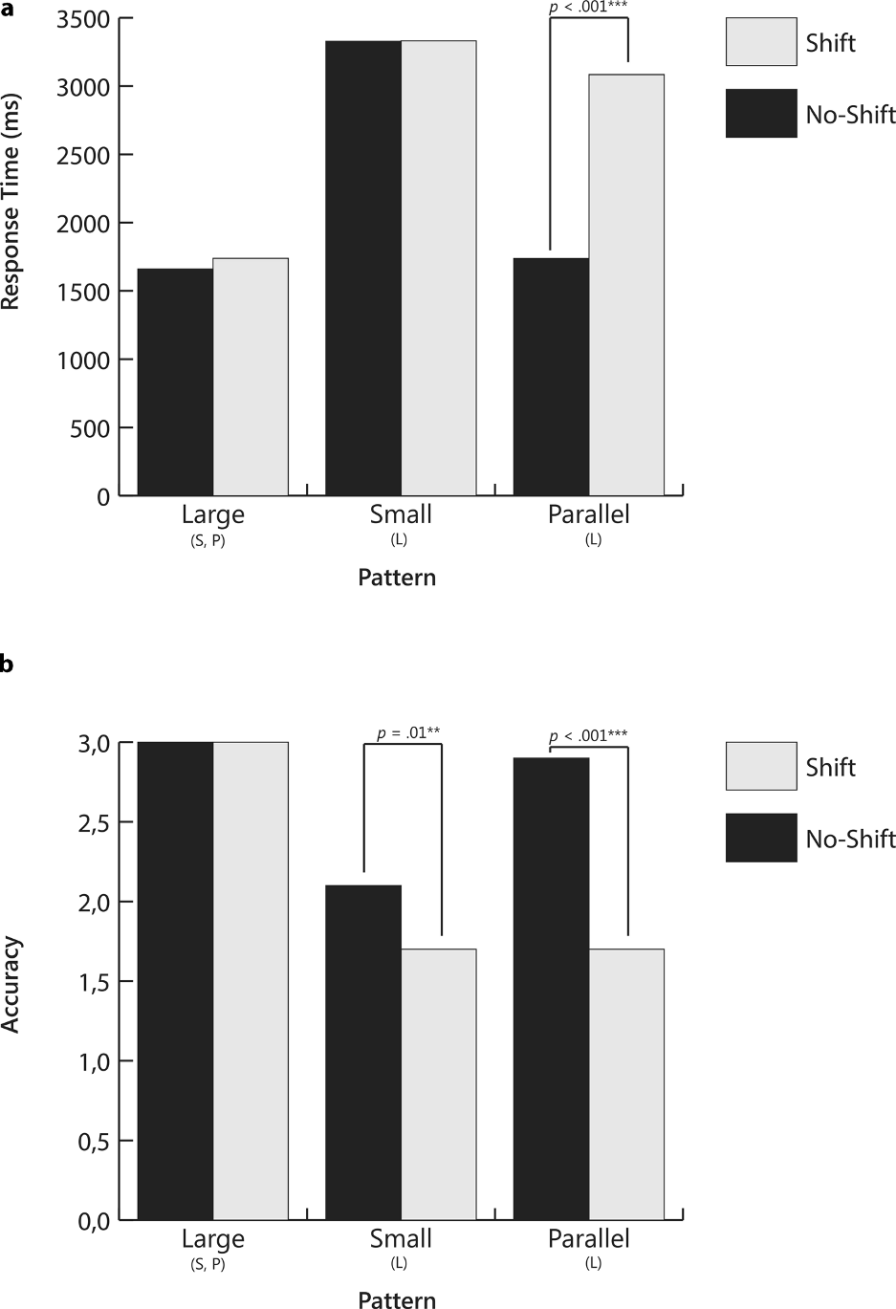
Mean response times and accuracy for slope-to-slope comparisons across slope difference and shift for positive slopes. Question was *Was the intervention effective?* Letters below bars signify significant differences between patterns: *L* large differences; *S* small differences; *P* parallel or no differences. *P-values* signify differences between shift and no-shift within pattern

**Fig. 8:**
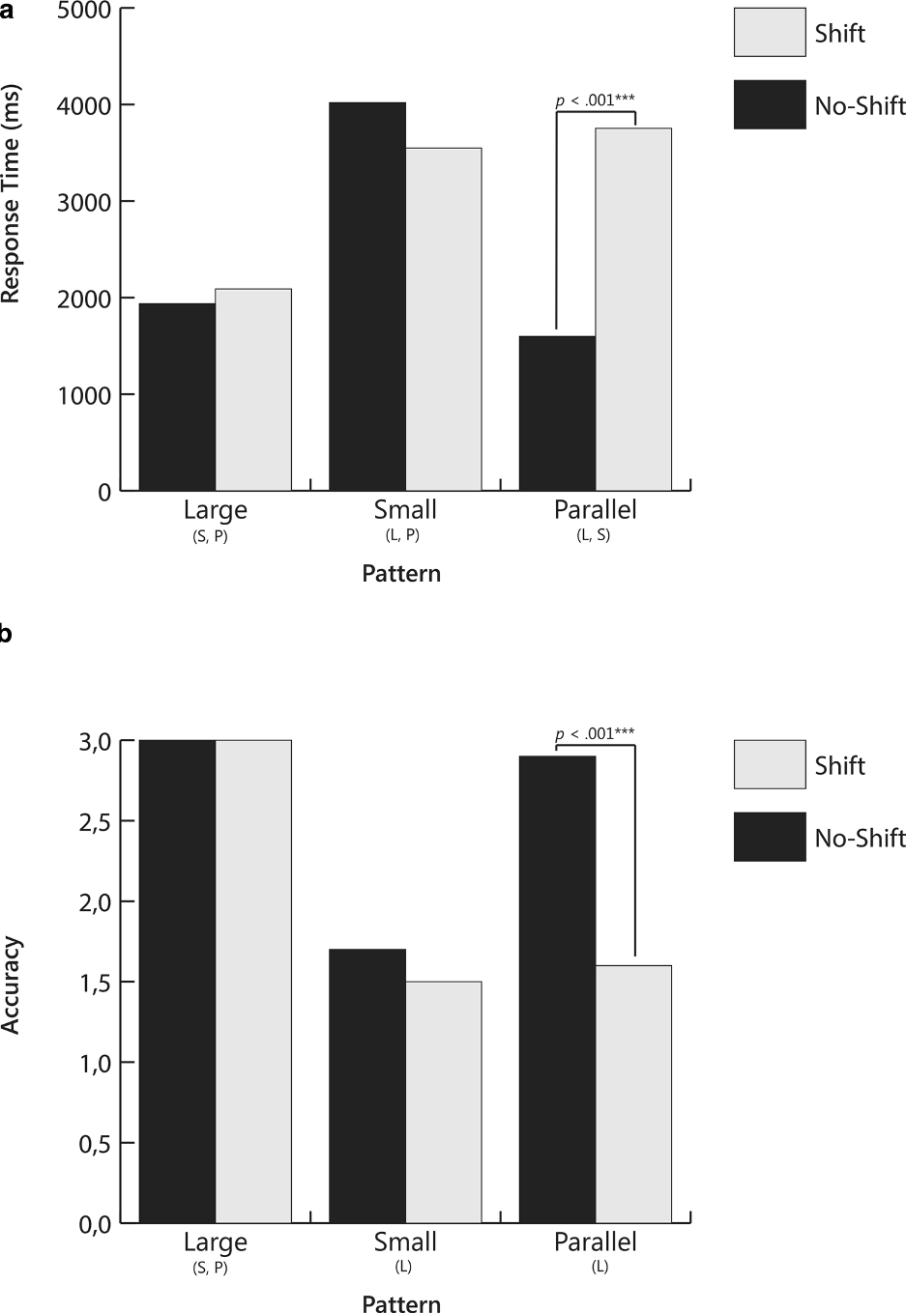
Mean response times and accuracy for slope-to-slope comparisons across slope difference and shift for negative slopes. Question was *Was the intervention effective?* Letters below bars signify significant differences between patterns: *L* large differences; *S* small differences; *P* parallel or no differences. *P-values* signify differences between shift vs. no-shift within pattern

Results of the MANOVA revealed an overall effect for direction (*V* = 0.26, *F*(2,28) = 4.85, *p* = 0.016, partial η^2^ = 0.26), slope differences (*V* = 0.58, *F*(4,116) = 11.72, *p* < 0.001, partial η^2^ = 0.29), and shift (*V* = 0.62, *F*(2,28) = 22.79, *p* < 0.001, partial η^2^ = 0.62). Results of the analysis are presented in Table 2 and displayed in Fig. 7 (positive direction) and Fig. 8 (negative direction).

**Table 2:** Means and Standard Errors for Response Time and Accuracy Across Slope Difference and Shift for Positive and Negative Slopes for Slope-to-Slope Comparison Question (Was the change in instruction effective?)

		Response Time				Accuracy			
	Shift	No Shift		Shift		No Shift		Shift	
Slope direction	Slope difference	M	SE	M	SE	M	SE	M	SE
Positive	Large	1660.21	146.44	1738.61	127.63	3.00	0.00	3.00	0.00
	Small	3328.58	382.07	3331.27	331.90	2.13	0.23	1.70	0.25
	Parallel	1737.66	127.45	3084.19	344.57	2.87	0.08	1.73	0.24
Negative	Large	1938.31	138.83	2090.64	133.01	3.00	0.00	3.00	0.00
	Small	4019.17	502.39	3549.78	370.01	1.73	0.25	1.47	0.24
	Parallel	1599.21	120.47	3753.41	464.05	2.87	0.10	1.57	0.27

Response times reported in milliseconds; Accuracy is the number correct out of 3

*M* Mean; *SE* Standard error

With regard to direction, follow-up ANOVAs revealed significant effects for response time (*F*(1,29) = 4.84, *p* = 0.036, partial η^2^ = 0.14) but not for accuracy (*F*(1,29) = 3.34, *p* = 0.078, partial η^2^ = 0.10). Participants showed longer response times when graphs displayed negative slopes (*M* = 2825.09, *SE* = 216.94) than when graphs displayed positive slopes (*M* = 2480.09, *SE* = 183.27).

With regard to slope differences, effects were significant for both response time (*F*(1.6,46.5) = 24.14, *p* < 0.001, partial η^2^ = 0.45) and accuracy (*F*(1.2,35.8) = 17.97, *p* < 0.001, partial η^2^ = 0.38). The multivariate interaction difference x shift (*V* = 0.59, *F*(4,116) = 12.08, *p* < 0.001, partial η^2^ = 0.29) was significant for both response time (*F*(2.0,57.6) = 24.75, *p* < 0.001, partial η^2^ = 0.46) and accuracy (*F*(1.2,34.2) = 11.51, *p* = 0.001, partial η^2^ = 0.28). Even though the multivariate effects of the interactions direction x difference (*V* = 0.59, *F*(4,116) = 12.08, *p* < 0.001, partial η^2^ = 0.29), direction x shift (*V* = 0.59, *F*(4,116) = 12.08, *p* < 0.001, partial η^2^ = 0.29), and the threeway-interaction direction x difference x shift (*V* = 0.59, *F*(4,116) = 12.08, *p* < 0.001, partial η^2^ = 0.29) were significant for both response time and accuracy, the univariate effects of these interactions were not significant.

Post-hoc tests with Bonferroni corrections were performed to test differences between slope conditions. Within both positive and negative direction conditions, response times were shortest and accuracy highest for graphs in which slope-value differences were large. In contrast, response times were longest and accuracy lowest when slope differences were small. For parallel slopes, response time and accuracy depended on whether the slope shifted. Response times were longer and accuracy lower when slopes shifted than when they did not shift. The only other pattern for which shift had an effect was in the positive direction condition for small slope differences; accuracy was lower when there was a shift in slope than when there was no shift.

In sum, data from the slope-to-slope comparison study reveal that the ease with which participants answered the question, *Was the change in instruction effective?* depended on the magnitude of the slope-value differences. When differences were large, graphs were easier to interpret; when differences were small, graphs were more difficult to interpret. When slopes were parallel, ease of interpretation depended on whether or not the slopes shifted. Accuracy and response times for parallel-shift patterns were similar to those seen for small-difference patterns.

## Discussion

The purpose of this study was to examine differences in ease of interpretation for various patterns of CBM graphed data. “Ease of interpretation” was measured via participants’ response times and accuracy when answering decision-making questions about the graph. We addressed one research question:* Are there differences in response times and accuracy when answering instructional decision-making questions about various patterns of CBM graphed data? *We hypothesized that ease of interpretation would vary with graph pattern, with some patterns being more difficult to interpret than others. Results confirmed our hypothesis: Response times and accuracy rates varied across graph patterns. Patterns for response times and accuracy did not always co-vary (i. e., longer response times, lower accuracy) confirming our decision to include both.

The study was a two-part study. In the first part, we examined the viewers’ ability to interpret the relation between the slope and goal line (*slope-to-goal)*. In the second part, we examined the viewer’s ability to interpret the relation between two adjacent slope lines (*slope-to-slope*). We discuss each part in turn.

### Slope-to-goal comparisons

Results of the *slope-to-goal* part of the study revealed differences in response times and accuracy rates across graph patterns, but differences were dependent upon the question to be answered. For both Questions 1 (*Will the student reach the goal?*) and 3 (*Should the teacher change the instruction?*), convergent graph patterns, in which the slope line converged upon the goal line (see Fig. 2d), appeared to be the most difficult to interpret. For both questions, accuracy was the lowest for convergent graphs, especially when the slope began above and converged upon the goal line. For Question 1, response times also were the longest for convergent graphs, indicating that these graph patterns were particularly difficult for participants in terms of deciding whether or not the student would reach the goal: It took participants longer to select an answer, and, despite the extra time, their answers were more often incorrect. Parallel patterns also proved somewhat difficult to interpret when the slope line began below the goal line. Although response times for these patterns were the same as, or only somewhat longer (Question 1) than, response times for the crossed and divergent patterns, participants’ answers to questions 1 and 3 were more often incorrect, suggesting that participants were confident in their interpretations, but their interpretations were incorrect. Divergent, crossed, and parallel-above patterns, in contrast, were easier to interpret, with relatively high accuracy and short response times.

Question 2 (*Did the intervention have a positive effect?*) revealed a different pattern of results than the other two questions. Responses to Question 2 were not influenced by the position of the slope, but, for crossed and divergent patterns, by the start point. Interestingly, the effect differed for the two patterns. For crossed patterns, interpretation was more difficult when the slope line began *above* and crossed under the goal line. Participants took longer to answer, and their answers were more often incorrect. For divergent graphs, it was more difficult when the slope line began *below* and diverged from the goal line. Although for this pattern, participants did not take longer to respond, they responded more often incorrectly, suggesting that participants were confident in their interpretations, but that the interpretations were incorrect.

Across the three questions, mean response times tended to decrease with each successive question, but accuracy did not show a corresponding increase. It is likely that inspecting the graphs to answer Question 1 made it possible to answer subsequent questions more quickly; however, this did not translate into answering the questions more accurately.

We cannot know for sure why some graph patterns proved to be more difficult than others to interpret, although the literature on graph comprehension provides one potential reason. Some patterns might be difficult to interpret because they require the viewer to make spatial transformations (Trickett and Trafton 2004). For example, to decide if the student will reach the goal (Question 1) for convergent graph patterns, the viewer must mentally extend the slope line and decide if and when it will cross the goal line. The goal is considered to be “reached” only if it crosses the goal line at or before the end point of the line. This spatial transformation might prove difficult, especially if the slope line would not cross until near the end point of the goal line.

A second potential reason that some graphs may be difficult is that they require the viewer to weigh the relative importance of level of performance vs. rate of growth, and the relative importance varies with graph pattern and question. For example, in parallel-below graph patterns, the student is progressing at the desired rate (the slope is parallel to the goal line), but is not performing at the desired level (the slope is under the goal line). To decide if the student will reach the goal (Question 1), the viewer must give more weight to level than to rate—the slope line will remain below the goal line and thus the student will not reach the goal. In this case, an incorrect interpretation of the graph pattern (student will reach goal) may lead to an erroneous instructional decision (do not change instruction).

In divergent-below patterns, the slope begins below and diverges from the goal line. The slope in these patterns might be in a positive direction—but less positive than desired, that is, less steep than the goal line. To decide if the intervention is having a positive effect (Question 2), the viewer must give more weight to rate than to level. The positive slope implies that the intervention is effective—the student is in fact growing. In this case, however, an incorrect interpretation (instruction is not having a positive effect) does not necessarily lead to an erroneous decision. The correct instructional decision is to change instruction because, even though the student is growing, the rate of growth is not fast enough for the student to reach the goal. The data reveal that participants did in fact have difficulty answering Question 2, but not Question 3, correctly (see Figs. 5 and 6).

The challenges associated with more difficult graph patterns could potentially be addressed via focused training and use of graphic aids. For example, our data suggest that it is important during CBM training to clarify the difference between rate of growth and level of performance, to explicitly teach when to emphasize one over the other, and to make clear that, even though an intervention may be having a positive effect on student growth, an instructional change might still be in order. With regard to graphic aids, our data suggest that interpretation of convergent or parallel-below patterns might be aided by graphically extending the slope lines to eliminate the need for spatial transformations. Likewise, for divergent-below patterns, displaying the graph with the slope line only (temporarily deleting the goal line) may make it easier to focus on the direction of the slope line in order to determine whether the intervention is having a positive effect. The goal line could then be added, and the slope line extended, to determine whether the instruction needs to be changed. The effects of such focused training/manipulations must be examined in future studies.

### Slope-to-slope comparisons

Results of the *slope-to-slope* part of the study revealed that, for both positive and negative slope conditions, interpretation was affected by slope-value difference and shift. In general, patterns with small differences between slopes, or with parallel slopes in which the second slope shifted, resulted in increased response times and lower accuracy rates, indicating that participants took longer to select their answers, but that their answers still were often incorrect.

Similar to the discussion about the *slope-to-goal *part of the study, one might surmise that small differences patterns are difficult to interpret because they are perceptually difficult to discern, and thus require spatial transformations for correct interpretation (Trickett and Trafton 2004). Inspection of the graphs displaying a small-difference pattern (Fig. 3c) reveals that, to decide if the instructional change is effective, the viewer must mentally “place” one slope on top of the other to determine which is steeper.

Also, similar to the previous discussion for the *slope-to-goal *patterns, parallel-shift patterns may be difficult to interpret because they require the relative weighing of level and rate. In parallel-shift patterns, the lines are parallel, but the second line shifts upward (see Fig. 3a). To decide if the change in instruction is effective, the viewer must give more weight to level than to rate. The upward shift implies that the intervention was effective, albeit perhaps only at the start. An incorrect interpretation may lead to the “erroneous” conclusion that the change was not effective, and the teacher may thus discontinue the new instructional program.

Our findings suggest that in CBM training, it is important to emphasize meaning of slope; that is, that it depicts the rate of growth within an instructional phase. In terms of the use of graphic aids, it may help the viewers to have the steeper slope highlighted in a different color. Again, the effects of such focused instruction and graphic aids must be examined in future research.

### The need to read beyond the data

Up to this point, we have focused primarily on two levels of graph interpretation: reading the data and reading between the data (Curcio 1987; Friel et al. 2001). However, as is perhaps evident, the “correct” decision in CBM graph interpretation ultimately rests with reading beyond the data. In our discussion, we have referred to “correct” interpretations and decisions; however, as mentioned earlier, the correct answers are in some cases ambiguous—and might perhaps even lead to different answers or longer response times among experts. For example, if the slope line is parallel to but below the goal line, should one conclude that the student is not “reaching” the goal? Likewise, if the slope line is positive, but less steep than and below the goal line, should one conclude that the intervention is having a “positive” effect?

To some extent, the answer to these questions depends on how one interprets the meaning of “reaching” the goal or having a “positive” effect. However, we would argue that the correct interpretation, and thus the correct instructional decision, ultimately hinges on the viewer’s ability to read beyond the data. The teacher must have knowledge of the literature and knowledge about the specific situation to arrive at a correct decision. Such decisions may be different than those dictated by standard decision-making rules.

With regard to knowledge of the literature, teachers should be made aware of, and take into account, the research on goal ambitiousness in a CBM system, which demonstrates that teachers who set more ambitious goals effect higher rates of student performance (see Stecker et al. 2005). With regard to knowledge of the specific situation, teachers should consider the students’ characteristics and the instructional setting. For example, if a student’s rate of growth is positive, but not positive enough to reach the goal, the teacher may decide to not change instruction if it seems that (1) the instructional approach is motivating to the student, (2) the student has difficulty with change, or (3) the approach has been shown in the past to take several weeks to exert an effect. In such cases, the teacher may decide to collect more data and then reevaluate growth.

### The importance of interpreting CBM graphed data

As a final point in the discussion, we would like to return to the issue of whether it is important to study interpretation of CBM graphs. After all, given that progress-monitoring programs could include computer-generated recommendations for instructional decisions, is there really a need to study the viewer’s interpretations of CBM graphs?

We believe that the answer is yes. First, as stated in the introduction, teachers often do not respond to the instructional recommendations provided by CBM graphing programs (Fuchs and Fuchs 2002). Second, the research presented here illustrates the importance of the practitioner in data interpretation and instructional decision-making: It is often necessary to read beyond the data to arrive at a correct interpretation. In sum, it is important to consider what the “active ingredient” in CBM progress monitoring is. Is it the data that are important, or is it the fact that implementation of a system such as CBM stimulates teachers to become *data-based instructional investigators *who continuously search for and evaluate approaches for building powerful and effective instructional programs for students who struggle? In future research, it will be important to search for the active ingredients in CBM implementation and data use, and in that regard, to examine the effects of data inspection and interpretation on data implementation and use.

### Limitations and future research

One potential limitation of the current study has already been discussed: For some graph patterns, different answers are possible. However, given that the purpose of the study was to examine differences in relative accuracy and response times among various graph patterns, the “true” answer to the question is less important than the extent to which respondents agreed with the researcher-selected answers across different patterns, and the relative amount of time they took to select their answers. Yet, for training purposes, it would be helpful to have agreed upon “correct” answers, which could be determined by asking experts to provide answers to the various patterns. Related to this suggestion, it would be interesting to replicate this study with experts to determine to what extent they agree upon the correct answers, and to examine whether certain patterns take longer to interpret than others for even the CBM experts. It would also be interesting in future research to interview the participants after they complete the tasks to ask why they selected the answers they did. This information could provide insight into what makes particular graph patterns difficult to interpret.

A second limitation of the study is the size and nature of the sample. With regard to sample size, the within-subjects design increased our power to identify differences, but it will be important to replicate the results. With regard to the nature of the sample, we purposefully recruited participants with no or limited knowledge and experience with CBM. It will be necessary, however, to replicate the study with teachers, especially with teachers who have experience implementing CBM. It may be that, with experience, some graph patterns become easier to interpret. In addition, it will be important in future studies to examine the influence of potential moderating factors, such as graph-reading skills, spatial skills, and knowledge of reading instruction.

A final limitation of the study is that we focused only on instructional-change decisions, excluding decisions related to raising the goal. We did not want to add yet another factor to the design, but decisions related to raising the goal should also be examined in future research, especially given the relation between goal ambitiousness and student achievement (see Stecker et al. 2005).

## Conclusions and implications

The results of this study demonstrate that there are differences in the ease with which various patterns of CBM graphed data can be interpreted. Two general conclusions can be drawn from our data. First, CBM graph patterns that are perceptually difficult to discern and that require spatial transformations are difficult to interpret. Second, and related to the first, viewers have difficulty taking into account both level of performance and rate of growth in interpreting graph interpretation.

Our findings have implications for training practitioners to use CBM. First, the results support the notion that direct training of graph interpretation is necessary. As stated by Glazer (2011), graph interpretation is complex and challenging. Our results imply that one cannot assume that all CBM graphs are easy to interpret. This research highlights which types of graph patterns are likely to be the most difficult to interpret, and thus which patterns should receive focused attention in training, or should be the targets of graphic aids. For example, it may be important to provide explicit instruction and multiple practice with patterns that require spatial transformations and that require consideration of both level and rate for interpretation. Likewise, including graph-reading aids, such as visually displaying extended slope lines in the graphs or highlighting the steeper slope, may improve the accuracy and efficiency of graph interpretation.

In conclusion, a focus on graph interpretation is important because it is not the collection and graphing of CBM data that leads to instructional improvements—it is the *use* of those data for making instructional decisions that leads to the creation of powerful and effective programs for students who struggle (Stecker et al. 2005).

## Caption Electronic Supplementary Material

Appendix A
