# Supplementary material for: Curriculum-Based Measurement progress data: Effects of graph pattern on ease of interpretation
Source: Z Erziehwiss. 2018 Jul 2;21(4):767–92. doi: 10.1007/s11618-018-0836-9 (PMC6428331; doi:10.1007/s11618-018-0836-9)
Supplement: Supplementary file 1 — Appendix A [file 11618_2018_836_MOESM1_ESM.docx]

**Appendix A**

**Instructions for Part 1: *Slope-to-goal* graph patterns**

Participants were shown a sample graph on the computer screen with a goal line and a slope line (similar to the graph displayed in Figure 2a). The researcher provided participants a description of the graph and of each element of the graph (see below). As each element was described, it was labeled on the graph on the screen. The instructions given to participants were as follows:

1. Here is a CBM graph. On the y-axis are reading scores and on the x-axis is time.
2. The baseline data represent the student’s beginning level of performance in reading and the peer line represents the level of performance for peers in the student’s class.
3. The goal line represents the student’s expected level of performance when provided with a particular reading intervention. The goal line extends to the end of Phase 2.
4. In the graph you also see a slope line. The slope represents the progress the student has made while receiving the reading intervention provided in Phase I. Depending on the effects of the intervention, the slope line will either increase or decrease. In the graph before you, the slope line is increasing.
5. The slope line stops at the end of Phase I because at this point the teacher must decide whether to continue with the intervention or not. (For purposes of this study, we assume that if the intervention in Phase 1 were to be continued, the rate of growth for the student would remain the same.)
6. To evaluate whether or not to continue the intervention, three questions must be answered: (1) Will the student reach the goal (if the intervention were to be continued)? (2) Did the instruction have a positive effect? (3) Should the teacher change the instruction?
7. To answer the three questions, you need to compare the slope line to the goal line. In the example, it can be seen that: (1) the student will achieve the goal; (2) the intervention has had a positive effect; (3) the intervention does not need to be changed.

Participants were then given five practice graphs. For each graph, they answered the following three questions: (1) *Will the student reach the goal?* (2) *Did the instruction have a positive effect?* (3) *Should the teacher change the instruction?*

**Instructions for Part 2: *Slope-to-slope* graph patterns**

Participants were shown a sample graph on the computer screen with two slope lines (similar to the graphs displayed in Figure 3). They were told that the graph displayed the progress of a student who was being taught by a teacher using a particular reading instructional approach, and that the set-up of the graph was similar to that of the graph in Part 1 except, that there were two slope lines, one in each phase. The researcher then provided a description of each element (see below), and as it was described, it was labeled on the graph on the screen. The instructions given to participants were as follows:

1. Part A represents the first phase of instruction for the student. The line in Part A represents the progress for the student during this time. (*Phase 1 is labeled with the letter A. Only the line in Part A is displayed on the graph.*)
2. At the end of the phase, the teacher makes a change in the instruction that the student is receiving.
3. Part B of the graph displays the student’s progress *after* the change in instruction was made for the student. (*Phase 2 is labeled with the letter B. Only the line in Part B is displayed on the graph.*)
4. The teacher now has to decide whether the change in instruction was effective for the student. The teacher uses the graph to answer this question. *(Both slope lines are now displayed on the graph.)*
5. In the example, the line in part B is increasing. The student’s progress is greater after the instructional change, thus the change in instruction has been effective. This is referred to as a *positive effect.* (*In the graph displayed to the participant, the slope in Phase 2 is positive and steeper than the slope in Phase I.)*
6. Here is an example in which the instructional change did not have a positive effect, but instead had a *negative effect.* Compared to Line A, Line B is less steep and decreases. (*In the graph displayed to the participant, the slope in Phase 2 is negative and less steep than the slope in Phase I.)*
7. In this final example, the instructional change resulted in *no* change in the student’s progress. In the example, Line B increases at the same rate as Line A. (*In the graph displayed to the participant, the slope in Phase 2 is parallel to the slope in Phase I.)*

Participants were then given five practice graphs. For each graph, they answered the following question, *Was the change in instruction effective?*
